# Enhancing Transforaminal Full Endoscopic Discectomy: Efficacy of the Hand Down Outside-In (HDOI) Technique

**DOI:** 10.3390/jpm14070679

**Published:** 2024-06-25

**Authors:** Yushi Yamaguchi, Koichiro Ono, Daisuke Fukuhara, Akira Dezawa, Tokifumi Majima

**Affiliations:** 1 Department of Orthopedic Surgery, Nippon Medical School, 1-1-5 Sendagi, Bunkyo-ku, Tokyo 113-8603, Japan; yushi-yamaguchi@nms.ac.jp (Y.Y.); d-fukuhara@nms.ac.jp (D.F.); t-majima@nms.ac.jp (T.M.); 2 Meiryu-kai Dezawa Akira PED Clinic, Tokyo 214-0014, Japan; adezawa@med.teikyo-u.ac.jp

**Keywords:** lumbar disc herniation, full endoscopic lumbar discectomy, transforaminal approach, hand down outside-in technique

## Abstract

Endoscopic lumbar discectomy (ELD) is a key advancement in minimally invasive spinal surgery, particularly for lumbar disc herniation. Interlaminar endoscopic lumbar discectomy (IELD) and transforaminal endoscopic lumbar discectomy (TELD) are the two major methods used for FED. TELD, while less familiar to spine surgeons inexperienced in endoscopic surgery, presents challenges in visualizing the dura mater, a crucial aspect for reducing surgical complications. The hand down outside-in (HDOI) technique introduced by Dezawa enhances this visualization by positioning the cannula tip dorsally on the intervertebral disc and maneuvering it between the dura mater and disc to the spinal canal’s midpoint. The cannula is then flipped to directly visualize the dura mater, enabling safe removal of the prolapsed disc material. A comparative study involving 20 patients treated from April 2020 to April 2022 examined the efficacy of the HDOI technique against conventional TELD. Each group, comprising ten patients, underwent ELD for lumbar disc herniation. While both groups showed similar improvements in clinical outcomes, as assessed using the Japanese Orthopedic Association (JOA) score and visual analogue scale (VAS) for pain, the HDOI group exhibited a 100% success rate for dura mater visualization, and this rate is significantly higher than the 60% observed in the conventional TELD group. Additionally, the time required for dura mater visualization was notably shorter for the HDOI technique. These results indicate that the HDOI technique not only enhances the safety and efficacy of TELD but may also encourage its wider use in clinical settings.

## 1. Introduction

Lumbar disc herniation, characterized by the posterior displacement of the nucleus pulposus, compresses the nerve root and dural sac in the lumbar spinal canal, eliciting significant limb and back pain. Surgical intervention is often considered for patients who do not respond to conservative treatments, particularly when symptoms include intolerable pain or neurological impairments such as motor dysfunction or autonomic disturbances [1,2]. While traditional open discectomy is known for its effective outcomes, the procedure’s invasiveness and associated complications often result in extended recovery periods [3]. Consequently, there has been a shift toward minimally invasive techniques to reduce surgical morbidity [4].

The evolution of full endoscopic lumbar discectomy (ELD) represents a significant milestone in the pursuit of less invasive spinal surgeries. Initially, percutaneous discectomy was introduced by Hijikata in 1975 as a precursor to endoscopic spinal surgery [5]. This approach was later enhanced by Yeung’s introduction of posterolateral endoscopic discectomy and Hoogland’s development of transforaminal posterolateral endoscopic discectomy [6,7]. In 2006, Ruetten further advanced these techniques by introducing interlaminar endoscopic lumbar discectomy (IELD) [8], contributing to the broader acceptance of ELD as a viable surgical option [9]. Minimally invasive techniques such as ELD offer numerous advantages, including reduced pain, minimal bleeding, lower risk of infection, and shorter hospital stays compared to traditional methods [10,11,12,13]. Transforaminal endoscopic lumbar discectomy (TELD) and interlaminar endoscopic lumbar discectomy (IELD) are the two major approaches to ELD [10]. Appropriate selection of these approaches allows for the effective removal of herniations located both outside and inside the spinal canal [10]. While each approach has unique benefits, IELD is particularly useful and frequently employed for diseases involving the L5/S1 region because of the large intervertebral space in the L5/S1 segment. Another advantage is that the surgical view is familiar to spine surgeons, as it is approached from the posterior side. TELD is especially noted for addressing various types of herniations, including paramedian, central, foraminal, and extraforaminal herniations [6,10]. TELD’s versatility has allowed its application to multiple pathologies, such as lumbar disc herniation, lumbar spine stenosis (LSS), metastatic tumors, discal cysts, and revision surgeries [3,9,11,14,15,16]. Despite its advantages, TELD is considered more technically challenging than IELD, particularly for surgeons inexperienced with endoscopic spine procedures [14]. The challenges include the unconventional visualization of the dura mater at the top side of the monitor display and differing traditional spine surgery setups. Another reason is the difficulty in visualizing the dura mater during TELD. Since the ELD procedure deals with soft tissue close to the dura mater, the inability to visualize the dura mater has a risk of injury. To mitigate these risks, Dezawa introduced the hand down outside-in (HDOI) technique in 2017, which enhances dural visualization and improves the safety of herniation resection [15]. This study evaluates the efficacy of the HDOI technique by comparing clinical outcomes between conventional TELD and TELD utilizing the HDOI method.

## 2. Materials and Methods

### 2.1. Study Design and Population

This retrospective study evaluated 20 patients who underwent transforaminal endoscopic lumbar discectomy (TELD) for symptomatic lumbar disc herniation at Nippon Medical School Hospital from April 2020 to April 2022. Patients were included if they had persistent symptoms despite at least six weeks of conservative treatment and were followed up for more than six months. Two techniques were compared: conventional TELD and the hand down outside-in (HDOI) method, with a transition to the HDOI technique occurring in April 2021.

### 2.2. Inclusion and Exclusion Criteria

The following inclusion criteria were employed: (1) lumbar disc herniation at levels L1/2 to L4/5; (2) prior conservative treatment for a minimum of six weeks; and (3) protruding, subligamentous, or transligamentous extruded disc herniations. The following exclusion criteria were used: (1) prior surgery at the affected disc level, (2) procedures involving fusion or laminectomy, (3) herniations at L5/S1, (4) extraforaminal herniations, and (5) presence of infection.

### 2.3. Ethical Considerations

The study adhered to the Declaration of Helsinki and was approved by the Ethics Committee and Institutional Review Board of Nippon Medical School (approval number: B-2022-622). After all participants were fully informed about the potential outcomes of the study, written informed consent was obtained.

### 2.4. Data Collection

Demographic and clinical data were extracted from medical charts by two authors (Y.Y. and K.O.). Magnetic resonance imaging (MRI) was used to assess herniation type, disc level, and laterality. A herniation is divided into protrusion (P), subligamentous extrusion (SE), transligamentous extrusion (TE), and sequestration (S) [17]. Other items were assessed from the patient’s medical records. Clinical outcomes were evaluated using the Japanese Orthopaedic Association (JOA) score and the visual analogue scale (VAS) for lumbar and lower limb pain at two weeks, one month, three months, and six months. The intraoperative video was reviewed to measure the duration and success of dura mater visualization, and postoperative complications were recorded from patient charts.

### 2.5. Surgical Techniques

All procedures were performed by a single surgeon (K.O.).

Conventional TELD technique

A spinal endoscope and other instruments (RIWOspine GmbH, Knittlingen, Germany) were used throughout the procedure.

**Figure 1:**
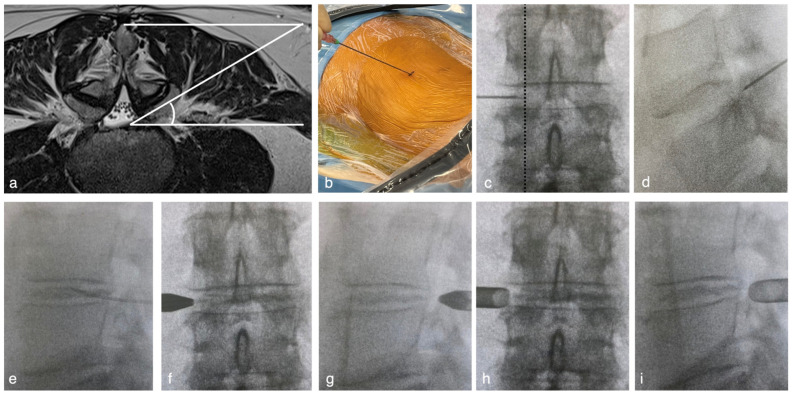
(**a**) The entry point is determined at 35° from the horizontal line based on the preoperative MRI. (**b**) After draping, under the A-P view of fluoroscopic guidance, a spinal needle is introduced from the entry point to the intervertebral disc, sliding the ventral aspect of the facet joint until the tip of the needle reached (**c**) the medial line of the facet joint (dotted line). (**d**) Change the fluoroscopic view to a lateral view and confirm that the tip is located at the posterior border of the vertebral disc. (**e**) Then, the needle proceeds into the intervertebral disc, and staining with Indigo carmine and imaging agent is performed. (**f,g**) A pencil dilator is inserted from the entry point until the tip of the dilator reaches Kambin’s triangle. (**h**,**i**) A bevel cannula is introduced over the pencil dilator.

**Figure 2:**
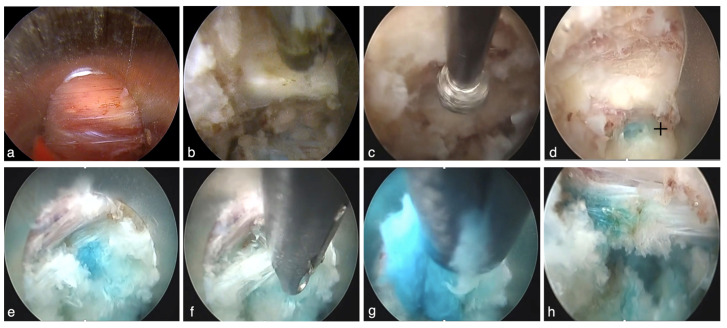
(**a**) A first endoscopic view. (**b**) Soft tissues are removed from a bipolar direction, and the lateral aspect of the superior facet is exposed. (**c**) The ventral aspect of the facet joint is drilled using a high-speed drill. (**d**) The blue-stained intervertebral disc (cross) is detected. (**e**) The intervertebral disc space is exposed. (**f**) The posterior annulus fibrosus is cut. (**g**) Resection of herniated disc material. (**h**) The removal of the herniation is complete, and the dura mater is not observed.

1. The patient was placed in a prone position under general anesthesia.
2. The entry point was determined to be 8–13 cm from the midline to acquire 45° from the horizontal line (Figure 1a). The spinal needles were inserted from the entry point until they reached the lateral aspect of the superior articular process (SAP) (Figure 1b). The needle tip was inserted into the foramen and slid along the ventral part of the facet joint (Figure 1c,d). Needle entry into the intervertebral disc should be more internal than the medial line of the cranial and caudal pedicles in the A-P and lateral views. The needle was advanced. The nucleus pulposus was stained with saline, indigo carmine, and an imaging agent (Figure 1e). Preoperative discography clarifies the difference between herniated fragments and other soft tissues under an endoscopic view.
3. The needle was replaced with a guidewire, and an approximately 3 cm incision was made at the entry point. A pencil dilator was then inserted along the guidewire (Figure 1f,g). The guidewire was removed after the tip of the pencil dilator touched the superior facet. A bevel-type cannula was inserted using a pencil dilator (Figure 1h,i). Finally, the endoscope was introduced.
4. Under endoscopic and fluoroscopic guidance, soft tissues were removed using a bipolar probe (Trigger-Flex Bipolar System; Elliquence, New York, NY, USA) and forceps (Figure 2a) to expose the lateral aspect of the facet joint and the superior articular process (SAP) (Figure 2b). After recognizing the bony orientation, the ventral part of the facet joint was drilled using a high-speed drill (Primado 2; NSK, Tokyo, Japan) (Figure 2c).
5. After detecting the blue-stained intervertebral disc (Figure 2d), a pencil dilator was inserted and hummed into the disc space. The cannula and endoscope were inserted again, and the herniated disc material was removed (Figure 2e–g).
6. After the procedure, the endoscope was removed. A drain tube was placed through the cannula as necessary, and the surgery was completed (Figure 2h).

TELD using the HDOI Technique [15]

**Figure 3:**
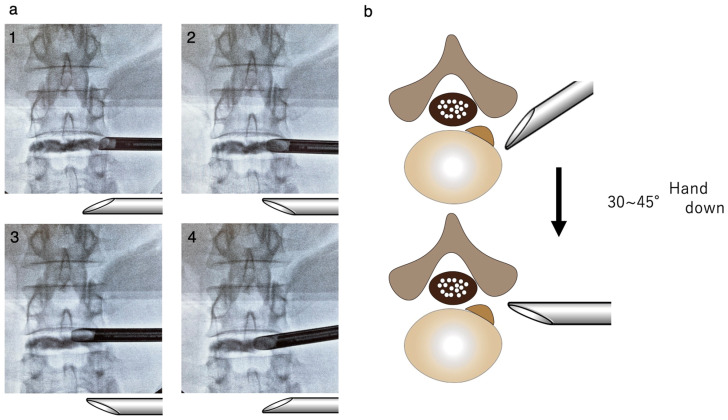
(**a1**) When the ventral part of the facet joint is drilled, the cannulas are upward. (**a2**) Change the tip of the cannula dorsally. (**a3**) Advance the cannula tip between the dura mater and the posterior aspect of the intervertebral disc. (**a4**) When the cannula reaches the herniation, it is reflipped, and the dura mater can be observed. (**b**) Place the cannula hand down 30 to 45 degrees from the horizontal line. Schematic diagram of how to move the cannula.

**Figure 4:**
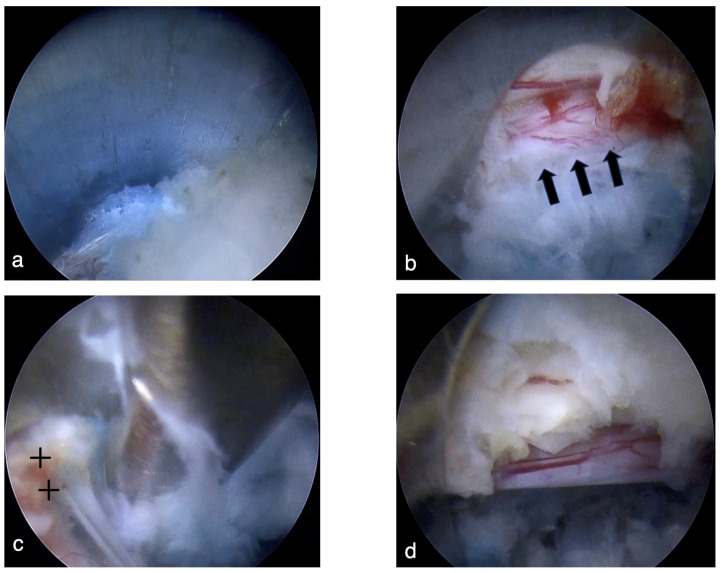
(**a**) After the blue-stained intervertebral disc is detected (Figure 2d), the cannula is flipped, placed on the tip dorsally, and advanced medially toward the herniation. It is important to press the cannula down to avoid losing the blue-stained disc during this maneuver. (**b**) Re-rotate the cannula, so the dura mater can be observed (short arrows). (**c**) Resection of the herniated disc material while confirming the dura mater (double cross). (**d**) The herniated material has been resected, and the loosening of the neural structures is confirmed.

Steps 1 through 4 of the HDOI technique were identical to the conventional TELD technique. The major difference in the HDOI technique is that in step 5, once the blue-stained disc was observed, the cannula was flipped and the tip was placed from ventral (Figure 3(a1)) to dorsal (Figure 3(a2)) and then advanced medially (outside-in) while gradually lowering the hand (hand down). The cannula was pushed towards the disc surface so as not to allow soft tissues, including the dura mater, to get into the cannula (Figure 3b and Figure 4a). Once the tip of the cannula reached the hernia (Figure 3(a3)), as confirmed by the A-P fluoroscopic image, the cannula was flipped again (Figure 3(a4)), and the ventral aspect of the dura mater was observed (Figure 4b). The posterior annulus fibrosus was cut with a micro-cutter if required, and the prolapsed herniation was safely removed while simultaneously observing the dura mater and the herniation (Figure 4c). After a sufficient amount of disc material had been removed and the floating of neural structures was observed, the endoscopic maneuver was done (Figure 4d).

Step 6 was also identical to that noted for the conventional TELD.

### 2.6. Statistical Analysis

Descriptive statistics were calculated for demographic and clinical variables. Means and standard deviations were used for continuous variables, and categorical variables were analyzed using the chi square test. A two-tailed Student’s *t*-test was employed for comparing continuous outcomes between the groups.

## 3. Results

This study enrolled 20 patients who met the inclusion and exclusion criteria: 10 underwent TELD using the hand down outside-on (HDOI) technique and 10 using conventional methods. The distribution of herniation levels and types across the two groups was as follows: at L1/2, L2/3, L3/4, and L4/5, herniation counts were 1/0, 1/4, 3/3, and 5/3 for the HDOI and conventional groups, respectively. Herniation types were categorized as protrusion (P), subligamentous extrusion (SE), transligamentous extrusion (TE), and sequestration (S), with occurrences of 0/1, 5/3, 5/6, and 0/0 in each respective category (Table 1). Hospital stay durations did not significantly differ between groups, averaging 1.7 days for the HDOI method and 2.2 days for the conventional technique. However, the follow-up period was significantly shorter for the HDOI group compared to the conventional group (8.3 vs. 14.4 months, *p* < 0.01) (Table 1).

**Figure 5:**
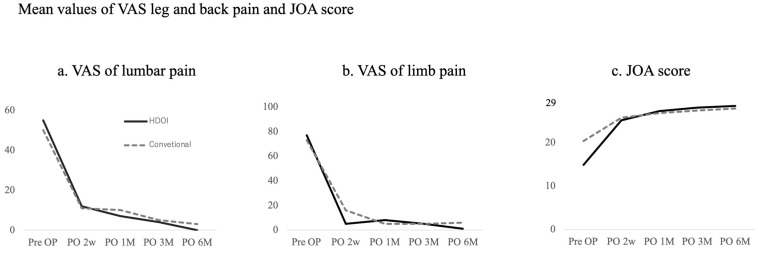
(**a**,**b**) VAS scores significantly improved equally for both procedures for back and lower limb pain. (**c**) JOA scores also improved equally for both procedures. JOA = Japanese Orthopedic Association; VAS = visual analog scale.

Clinical outcomes assessed using the Japanese Orthopaedic Association (JOA) score showed equal improvement post-procedure in both groups. The visual analogue scale (VAS) for back and lower limb pain also showed significant improvement, as illustrated in Figure 5. Visualization of the dura mater was achieved in 100% of patients in the HDOI group compared to 60% in the conventional group, with this difference being statistically significant (*p* = 0.025) (Table 2). The time required to visualize the dura mater from the onset of the endoscopy was significantly shorter in the HDOI group than in the conventional group, averaging 16.5 min versus 31.5 min (*p* = 0.045). Additionally, the mean operation time tended to be shorter in the HDOI group than in the conventional group; however, this difference approached but did not reach statistical significance (59.9 vs. 69.2 min, *p* = 0.058). In terms of complications, intraoperative dural injury occurred in one patient in the conventional group, with no such incidents reported in the HDOI group. No further complications were noted in either group.

## 4. Discussion

This observational comparative study demonstrated that the HDOI technique consistently achieved 100% visualization of the dura mater without any instances of dural injury. TELD typically employs two primary methods: the inside-out and outside-in techniques [7,16]. The inside-out technique is performed through Kambin’s triangle by inserting a dilator directly into the intervertebral disc space and placing a cannula and endoscope [18]. Therefore, the nuclear pulposus is observed at the beginning of the endoscopic procedure [18]. This method is straightforward and particularly beneficial for spine surgeons inexperienced with endoscopic techniques due to its minimal requirement for soft tissue manipulation. However, challenges arise in cases with a narrow foramen where retraction of the exiting nerve root may lead to dysesthesia due to irritation of the dorsal root ganglion, rendering this method unsuitable. Conversely, the outside-in technique, preferred for such cases, involves enlarging the foramen through the removal of the ventral aspect of the superior articular process (SAP) using a high-speed drill. This approach allows for more space between the exiting nerve root and the endoscope, facilitating safer discectomy despite being technically demanding due to the required soft tissue resection. Nonetheless, this technique has been associated with higher reoperation rates [10], partly due to difficulties in directly visualizing the dura mater. To address these challenges, Dezawa developed the HDOI technique. During the HDOI procedure, after enlarging the foramen and confirming the blue-stained intervertebral disc, the cannula is flipped, and the tip of the cannula is placed dorsally and advanced medially between the dura mater and the intervertebral disc while observing the intervertebral disc. On reaching the midline, the cannula is turned so that the dura mater could be observed. Foramen enlargement is required if the cannula cannot be inserted. Finally, discectomy can be performed with recognition of the dura mater. The risk of dural injury is considered to be as low as possible with this technique, as the hernia resection is performed with theoretical visibility. Thus, the HDOI technique is useful for the transforaminal technique and has a gentle learning curve. Dural injury, a significant complication of TELD, is recorded at a rate of 0.4% in the literature [19]. Minor lacerations without neural entrapment syndrome or nerve root exposure often heal spontaneously or with conservative management such as fluid infusion, while more substantial injuries may require open repair with dural stitches and shielding materials [19]. In our study, the conventional outside-in TF approach failed to visualize the dura mater in 40% of cases, with one incident of dural injury that fortunately did not necessitate open conversion for repair. However, if the cauda equina overflows due to a large laceration, endoscopic repair might be difficult, and the injury may require open conversion for repair. In stark contrast, the HDOI technique accomplished a 100% visualization rate of the dura mater with no dural injury, underscoring the technique’s enhancement of surgical safety by allowing continuous monitoring of the dura mater during discectomy. None of the existing literature reported dural visualization for surgical procedures using conventional approaches. Therefore, it is not possible to compare visualization with previous studies, but the results of the present study predict that the HDOI method has a higher visibility rate than the conventional method. Additionally, the time to visualize the dura mater was significantly shorter in the HDOI group compared to the conventional method, facilitating more precise and quicker herniation resections. Consequently, the surgeon could perform a more accurate hernia resection, and the operative time tended to be shorter in the HDOI group than in the conventional technique group (*p* = 0.057). Our overall operation times for TELD were longer than previously reported [10], and this is likely due to the inclusion of steps such as intervertebral disc staining and drain tube placement. Additionally, our facility is an academic medical center, and the staff involved in TELD, including nurses and radiological technologists, are not part of the same dedicated team. More specifically, there are occasional situations where surgery time is wasted due to poor timing in replacing the perfusion water system and a lack of accurate C-arm manipulation techniques. These factors might have resulted in the extended operation times compared to those in the previous study. However, these times may decrease significantly with increased procedural familiarity and case volume. There are two possible reasons for the longer hospital stays compared with other countries. The first is that all patients had a drain placed that is removed after several days, prolonging their hospital stay. The second reason is Japan’s national health insurance system, which lowers the necessary costs for hospitalization compared to Europe and the USA. This likely contributes to the longer hospitalization periods. Many studies have described clinical outcomes using the VAS, JOA, and Oswestry low back pain disability questionnaire (ODI) to assess TELD [10,19]. Therefore, we used a similar method to evaluate clinical outcomes. All these studies had good clinical outcomes and reported results identical to ours. The reason for this may be that there were no differences in height or type between the two groups. The exclusion of cases unsuitable for TELD, such as those at L5/S1, may have led to good postoperative outcomes in both groups. However, most reports set a follow-up period of approximately two years, whereas our report had a short period of six months. Therefore, long-term performance must be evaluated. The efficiency and safety of TELD could be greatly enhanced by standardizing the use of the HDOI technique. Future goals are to further increase the number of cases and consider the clinical results.

## 5. Limitation

This study has several limitations. First, this study is subject to all the limitations of a retrospective analysis including a limited sample size and selection bias. Second, this study is subject to measurement inaccuracies, both in clinical and radiographic assessments. In the future, the number of patients should be increased, and further studies should be conducted.

## 6. Conclusions

We clarified that the HDOI technique can visualize the dura mater in all cases without dural injury compared to the conventional technique. The herniated disc material was safely removed when visualizing the dura mater using the HDOI method. Favorable clinical outcomes indicated that the HDOI technique could facilitate the TF approach.

## Figures and Tables

**Table 1:** Patient demographics and clinical data.

Characteristic	HDOI	Conventional	*p*-Value
Case number	10	10	
Age (range)	62.7 (20–82)	59.9 (40–88)	0.73
Sex (male/female)	9/1	6/4	n.s.
Surgical levels (Ll/2, 2/3, 3/4, 4/5)	1, 1, 3, 5	0, 4, 3, 3	n.s.
Type of herniation (P, SE, TE)	0,5,5	1,6,3	n.s.
Postoperative hospitalization time (days)	1.7	2.2	0.23
Follow-up periods (month)	8.3	14.4	*p* < 0.01

**Table 2:** Reported outcomes after surgery comparing the HDOI and conventional techniques.

	HDOI	Conventional	*p* Value
Operation time (mins)	59.9	69.2	0.058
Time for dura recognition (min)	16.5	31.5	0.045 *
Recognition rate (%)	100	60	0.025 *
Complication (case no.)	0		n.s.

* *p* < 0.05.

## Data Availability

The data used to support the funding of this study are available from the corresponding author upon request.
